# Contrasting Roles for Orbitofrontal Cortex and Amygdala in Credit Assignment and Learning in Macaques

**DOI:** 10.1016/j.neuron.2015.08.018

**Published:** 2015-09-02

**Authors:** Bolton K.H. Chau, Jérôme Sallet, Georgios K. Papageorgiou, MaryAnn P. Noonan, Andrew H. Bell, Mark E. Walton, Matthew F.S. Rushworth

**Affiliations:** 1 Department of Experimental Psychology, University of Oxford, OX1 3UD, Oxford, UK; 2 Department of Psychology, The University of Hong Kong, Pokfulam Road, Hong Kong; 3 MRC Cognition and Brain Sciences Unit, 15 Chaucer Road, Cambridge, CB2 7EF, UK

## Abstract

Recent studies have challenged the view that orbitofrontal cortex (OFC) and amygdala mediate flexible reward-guided behavior. We trained macaques to perform an object discrimination reversal task during fMRI sessions and identified a lateral OFC (lOFC) region in which activity predicted adaptive win-stay/lose-shift behavior. Amygdala and lOFC activity was more strongly coupled on lose-shift trials. However, lOFC-amygdala coupling was also modulated by the relevance of reward information in a manner consistent with a role in establishing how credit for reward should be assigned. Day-to-day fluctuations in signals and signal coupling were correlated with day-to-day fluctuation in performance. A second experiment confirmed the existence of signals for adaptive stay/shift behavior in lOFC and reflecting irrelevant reward in the amygdala in a probabilistic learning task. Our data demonstrate that OFC and amygdala each make unique contributions to flexible behavior and credit assignment.

## Highlights

• Orbitofrontal cortex determines future behavior on the basis of reward feedback
• Variation in orbitofrontal cortex activity is correlated with variation in learning
• Amygdala carries information about irrelevant reward
• Amygdala-orbitofrontal interactions emphasize relevant not irrelevant reward

## Teaser

Chau et al. identify a posterior lateral OFC (lOFC) region in which activity predicts adaptive win-stay/lose-shift behavior. Amygdala activity predicts only lose-shift behavior but carries information about irrelevant rewards. lOFC-amygdala connectivity is modulated dynamically by the relevance of reward information.

## Introduction

The orbitofrontal cortex (OFC) together with the amygdala have long been thought to mediate changes in behaviors, particularly those that are guided by changes in the reward environment (Aggleton and Passingham, 1981; Butter, 1969; Izquierdo et al., 2004; Jones and Mishkin, 1972; Morrison et al., 2011; Paton et al., 2006; Roberts, 2006; Rudebeck and Murray, 2008; Spiegler and Mishkin, 1981). In experimental settings, such behavioral change has often been studied in the context of object discrimination reversal (ODR) tasks in which reward is first assigned to one stimulus but not another. After animals reach a high level of responding to the rewarded stimulus, the reward assignment is switched and the reversal occurs; the previously rewarded stimulus is no longer rewarded and the previously unrewarded stimulus is now rewarded.

The links between OFC, amygdala, and reward-guided behavioral change have recently been challenged. Rudebeck and colleagues (2013b) reported that OFC lesions do not cause macaques to perform poorly on reversal tasks if the lesion is made by neurotoxin injection rather than by aspiration, suggesting that the reversal task impairments seen after OFC lesions are actually the consequence of deafferentation of an adjacent brain region but not of damage to the OFC per se. The identity of the critical brain region is unknown. Although OFC neuron activity has been reported during stimulus-reward reversal learning (Morrison et al., 2011; Rolls, 2000), it might be argued that had another frontal region been investigated then the proportion of neurons encoding critical task variables might have been higher.

A similar challenge has been mounted on the idea that the amygdala is important for ODR. Neurotoxic lesions in the amygdala have been reported not to affect (Izquierdo and Murray, 2007) or even to improve ODR performance (Izquierdo et al., 2013; Rudebeck and Murray, 2008).

Adding to the confusion are observations that while neurotoxic lesions do not cause reversal impairments in an old world primate like the macaque, they can impair reversal tasks in rodents (Schoenbaum et al., 2007) and new world monkeys such as marmosets (Roberts, 2006). However, even in some of these species the role of the amygdala has been questioned. Stalnaker and colleagues (2007) showed that the reversal impairments caused by bilateral OFC lesions in the rat were abolished by bilateral lesions of the amygdala. The results suggest the surprising conclusion that OFC, at least in the rat, is counteracting or suppressing some feature of amygdala activity that disrupts rapid reward-guided behavioral change. Such a conclusion, however, is at odds with other claims based on neurophysiological data that amygdala and OFC carry related signals at short latency differences suggestive of inter-areal interaction and collaboration and the exchange of information during behavioral change (Morrison et al., 2011).

We attempted to reconcile these conflicting findings by recording activity throughout the whole brain using fMRI in four macaque monkeys while they performed a deterministic ODR task (experiment 1) and a probabilistic version of the ODR task (probabilistic learning task: experiment 2). In this way, we hoped to identify frontal cortical regions carrying signals needed for reward-guided behavioral reversal in an old world primate in which behavioral change cannot be mediated by verbal or linguistic strategies that in human will depend on adjacent ventrolateral frontal cortical regions associated with language. In addition, because we recorded activity from the whole extent of OFC and amygdala simultaneously from all four animals, we could examine interactions between OFC and amygdala. In brief, we identify a region extending from anterior insula to area 12/47 that we refer to as lateral OFC (lOFC) that lies just outside the focus of many OFC investigations in the macaque that carries signals needed for behavioral change. We also found behavioral change signals in amygdala distinct to those in lOFC. We also identified two distinct types of lOFC-amygdala interaction that might be labeled positive and negative and that might be linked to exchange and suppression of signals respectively. We contend that the interaction between lOFC and amygdala changes dynamically to ensure accurate stimulus-reward credit assignment and to avoid the integration of irrelevant reward information in estimates of stimulus-reward association.

## Results

### Animals’ Behavior

**Figure 1:**
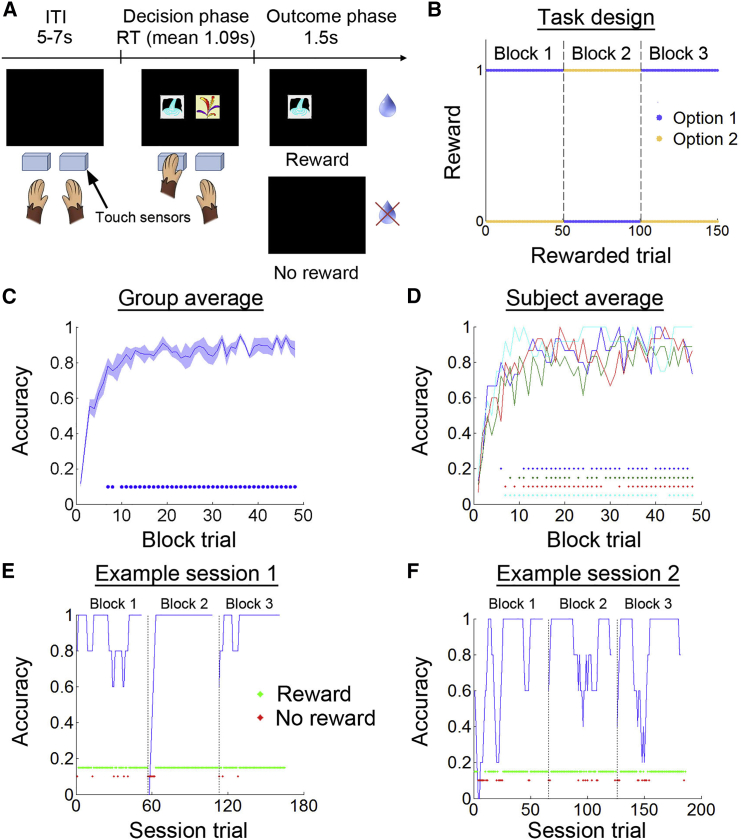
Object Discrimination Reversal Task (A) Each trial started with an inter-trial interval (ITI) showing a blank screen. Two options were then presented on the screen, monkeys chose one of the options by reaching the touch sensor placed in front of it (decision phase). Juice reward was delivered if a correct option was chosen (outcome phase). (B) The task was designed with a two-option deterministic reversal schedule. Each session began with one correct option that led to a reward and one incorrect option that did not lead to a reward. The stimulus-reward contingencies reversed after monkeys performed 50 and 100 rewarded trials. (C) On average, the accuracies of all monkeys were low on the early trials in a block and gradually increased. (D) Performance averaged across testing sessions within subject. Each line represents data from one subject. The raster plots in (C) and (D) indicate trials in a block with accuracies significantly higher than 0.5 (p < 0.05). (E and F) Example sessions from two different subjects. Accuracies were calculated by using a moving average window of 5 trials. The dotted lines indicate reversals. The raster plots indicate the reward (green) and no reward (red) outcome events.

Four monkeys performed the deterministic ODR task (experiment 1; Figure 1A); they had to discriminate which of two options led to a reward at the beginning of a session. The reward assignment reversed after the monkeys performed the 50^th^ rewarded trial and then it reversed again after the 100^th^ rewarded trial. The daily session stopped after the monkey performed 150 rewarded trials in total (Figure 1B). The animals encountered new stimuli at the beginning of each day of testing. On average, the monkeys performed 183.4 trials (81.8% correct) in each session and each monkey contributed four to six sessions in this dataset. To investigate the animals’ behavior, we split each session into three blocks (block 1: the initial learning period before any reversals; blocks 2 and 3: after the first and second reversal, respectively) and calculated the average accuracy of each trial as a function of its position in the block (trial 1, trial 2, trial 3, and so on; Figure 1C). The monkeys typically showed poor accuracy on early trials but they were consistently above 50% correct after the ninth trial of a block (*t*_*3*_ > 3.920, p < 0.030; Figure 1C). When block 1 and blocks 2 + 3 were analyzed separately, the first trials of block 1 had a higher accuracy (38%), due to random decisions, than comparable trials in blocks 2 + 3 (0%) due to post-reversal decisions (Figure S1). The average performance of each monkey is shown in Figure 1D. To illustrate performance on individual testing sessions, the data were smoothed by calculating a running average over five trials that stopped at the last four trials of a block to avoid inclusion of trials in different blocks within a given average. Figures 1E and 1F show monkeys’ performances in two example sessions.

### lOFC Encoded a Win-Stay/Lose-Shift Signal

**Figure 2:**
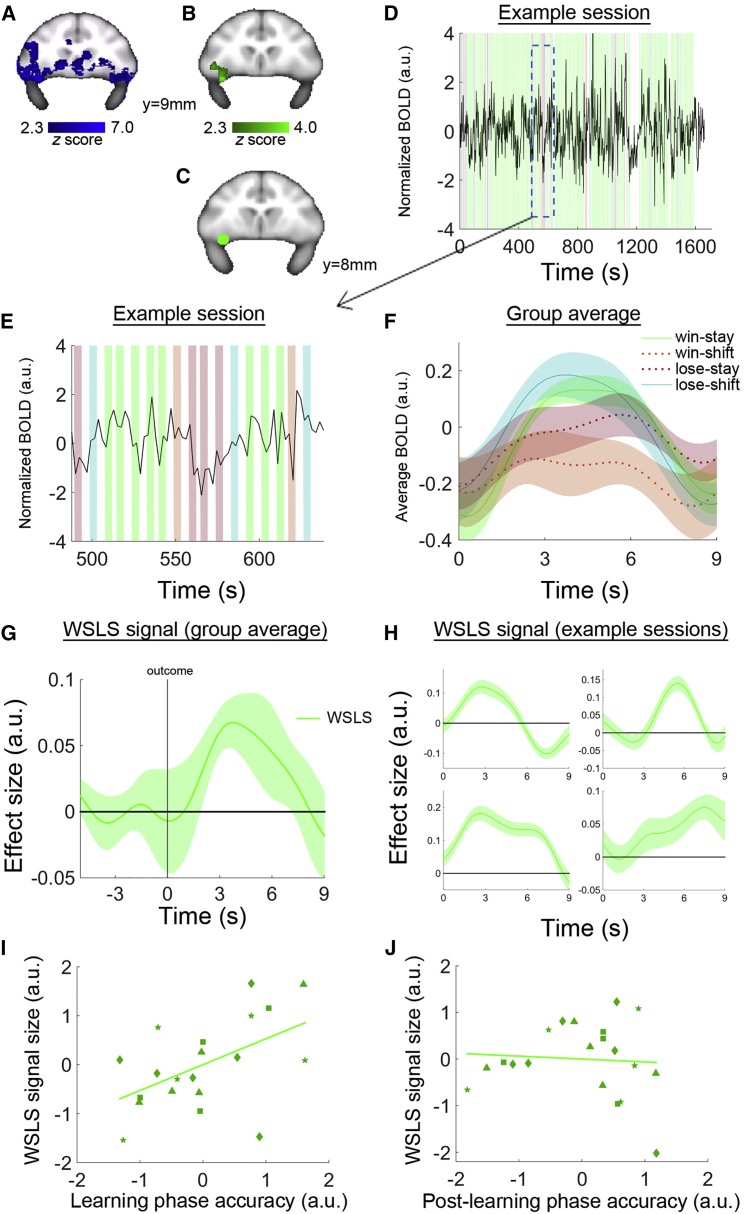
Win-Stay/Lose-Shift Signal in the lOFC (A) A whole-brain analysis showing a signal in the lOFC that was related to the occurrence of an outcome event. (B) A whole-brain analysis showing a signal in the lOFC that predicted win-stay/lose-shift behavior. (C) lOFC (16, 8, −4; green) BOLD activity was extracted for ROI analysis. (D and E) BOLD signal time course in the lOFC from an example session. The task events of win-stay, win-shift, lose-stay, and lose-shift are labeled in green, orange, red, and blue, respectively. (F) The BOLD signal was time locked at the outcome phase of the task and averaged across testing sessions and subjects. (G) The lOFC showed WSLS activity that ramped up after the onset of the outcome phase and peaked at around 4 s (green). (H) All four subjects consistently showed a WSLS signal after the outcome was revealed at 0 s. (I) The signal was extracted from the time window indicated by the bracket above the time course (which corresponds to the full-width half-maximum of the peak established using a leave-one-out procedure) and correlated with behavior. Testing sessions with larger WSLS signals in the lOFC were related to higher accuracies during the learning phase (first 9 trials in a block). (J) The sizes of the WSLS signal had no relationship with accuracies in the post-learning phase (after 30 trials in a block). Each type of marker symbol in (I) and (J) represents data from one animal.

In this task, an optimal strategy is to stay with the same choice on the next trial after rewarded decisions but to shift to the alternative choice after non-rewarded decisions. In other words, monkeys should make use of the outcome feedback and follow a win-stay/lose-shift (WSLS) rule for guiding their behavior. Our first analysis, therefore, examined activity across the whole brain to identify regions that were sensitive to the occurrence of the outcome event of the task, regardless whether the outcome was a reward or not, using standard fMRI blood-oxygen-level-dependent (BOLD) imaging analysis procedures (see Experimental Procedures). Two example BOLD data volumes and mean BOLD data from two example sessions are shown in Figure S2A. We found that, bilaterally, lOFC became more active when the choice outcome was revealed (cluster-based thresholding *z* > 2.3, p < 0.05 cluster-corrected; Figure 2A). The lOFC signal was consistently found in all four animals (although in one subject the signal only exceeded the conservative threshold for significance in one hemisphere; Figure S2b). In addition, outcome-related activation was found in a number of other areas (Table S1).

Next, we investigated how the outcome feedback was used to guide adaptive behavior on the next trial by conducting the key whole-brain analysis to search for regions encoding the WSLS rule. It has been suggested that OFC is not critical for reward-guided behavioral change (Rudebeck et al., 2013b) and by carrying out this analysis we hoped to identify which adjacent frontal area, if any, might possess activity related to the deployment of the WSLS rule needed for reward-guided behavioral change. The effect of the WSLS rule on neural activity can actually be thought of as the effect of an interaction term on brain activity where the main effects are of “reward delivery” (versus non-delivery) and “choice shifting” (the next selected option is different from the current trial, versus a stay choice, where the next selected option is the same as on the current trial). We therefore also included these two main effects in our regression model. Whether reward was delivered or not had a broad impact on activity throughout the brain as has been previously reported (Vickery et al., 2011) and so, for the sake of clarity, we focus on WSLS in the following fMRI analyses because these effects varied across regions and because they are the signals most directly related to the guidance of future behavior.

We identified a region in lOFC, which overlaps with part of the lOFC cluster that was active during the outcome events in general, that carried a signal guiding WSLS behavior on the next trial (cluster-based thresholding *z* > 2.3, p < 0.05 cluster-corrected; Figure 2B; Table S1; Figure S3). The activation lay in a relatively posterior location and just lateral to the lateral orbital sulcus and therefore just outside the region affected by the neurotoxic lesions made by Rudebeck and colleagues (2013b). It is therefore likely that the connections of this region would have been affected by the aspiration lesions that Rudebeck and colleagues showed did compromise reversal task performance.

To illustrate the significant activity in lOFC, we placed a region of interest (ROI) over the peak of the lOFC effect (Figure 2C) and extracted the time course of the BOLD data (Figures 2D and 2E). We avoided “double dipping” (Kriegeskorte et al., 2009) as we did not conduct further statistical analysis at this stage. A WSLS effect is manifested when the BOLD signal for trials of win-stay/lose-shift is greater than for trials of win-shift/lose-stay. This was the case in lOFC when we time locked the BOLD data to the onset of the outcome phase (Figure 2F). When we directly compared win-stay/lose-shift and win-shift/lose-stay events in lOFC, there was a WSLS signal that ramped up after the onset of the outcome phase and peaked with a delay of around 4 s (Figure 2G). The latency is similar to that seen in other macaque BOLD imaging studies (Leite et al., 2002). The WSLS signal was similar across animals (Figure 2H).

The whole-brain fMRI test that we conducted follows the most widely used conventions in neuroimaging experiments. These conventions, however, are designed to deal with the difficulty of performing mass univariate statistical comparisons across multiple MRI voxels and are known to be conservative. It is well known, however, that such an approach can fail to identify meaningful and replicable effects especially when their spatial extent is limited. We therefore used an additional ROI approach to examine the relationship between BOLD and WSLS in the central OFC region between the medial and lateral orbital sulci where neurotoxic lesions were made in previous studies. The WSLS effect in that region did not reach statistical significance (*t*_3_ = 0.503, p = 0.650; Figure S4A). Because of the conservative nature of the whole-brain statistical test that we used to identify activity in right lOFC, we were concerned that there might have been similar activity in left lOFC too. We therefore tested whether the WSLS signal in the lOFC was lateralized by placing an ROI in the contralateral lOFC. There was a significant WSLS signal in the left lOFC (*t*_3_ = 15.915, p = 0.001; Figure S4B) and the strengths of the signals were not significantly different between the two hemispheres (*t*_3_ = 0.464, p = 0.674).

In summary, while there are neurons in the central OFC region that are important for WSLS behavior, the lOFC may be especially important when reward outcomes are used to guide adaptive behavior. To investigate whether this was actually the case, we next tested how the lOFC WSLS signal was related to task performance. Note that this test focuses on the fact that the strength of the WSLS signal and task performance varied session by session and therefore this test is statistically orthogonal to the original test used to identify the WSLS signal. We obtained the peak size of the WSLS signal from each animal on each testing session and the average choice accuracy from each animal on each testing session. Since the testing sessions were contributed by four different monkeys (four to six sessions per monkey), we normalized the peak lOFC signal sizes and the behavioral accuracy measurements within each individual animal to remove between subject variance (Supplemental Experimental Procedures).

In the behavioral task, learning the option values was important during the early trials of a block (learning phase: in the first nine trials of each block before the group accuracy [Figure 1C] was consistently higher than 50%), whereas maintaining high choice consistency or low stochasticity was crucial for the monkeys after the learning phase (post-learning phase: after 30 trials of each block when group accuracy was consistently higher than 70%). Similar definitions of learning stages were previously employed in reversal learning studies (Jones and Mishkin, 1972; Murray and Izquierdo, 2007). We found that testing sessions with stronger lOFC WSLS signals were associated with higher accuracies in the learning phase even after controlling for accuracy in the post-learning phases (*r* = 0.518, p = 0.023; Figure 2I). The lOFC WSLS signal was not, however, related to accuracy in the post-learning phase once accuracy in the learning phase was controlled (*r* = −0.071, p = 0.773; Figure 2J). These conclusions were confirmed when a complementary reinforcement learning model was applied (Supplemental Experimental Procedures); the lOFC WSLS signal was positively related to the learning rate parameter and unrelated to the stochasticity parameter (Figure S5).

### Comparing the Roles of lOFC and Amygdala in Guiding Future Behavior

There is uncertainty about the role of primate amygdala in stimulus-reward reversal learning. Aspiration and radiofrequency lesions impair stimulus-reward reversal learning (Aggleton and Passingham, 1981; Jones and Mishkin, 1972; Spiegler and Mishkin, 1981) but such lesions also compromise fibers of passage through the amygdala and subjacent cortex. Confusingly neurotoxic lesions of the amygdala in rats have been reported as impairing (Schoenbaum et al., 2003) and improving (Izquierdo et al., 2013) stimulus-reward association learning, while in old world monkeys they have been reported as leaving ODR performance unaffected (Izquierdo and Murray, 2007) or even improved (Rudebeck and Murray, 2008).

Some amygdala lesion-induced changes in OFC activity and other neurophysiological recordings made in the OFC and amygdala suggest that the regions exchange information in order to bring about reward-guided behavioral change (Morrison et al., 2011; Rudebeck et al., 2013a; Saddoris et al., 2005). By contrast, the fact that OFC lesion-induced impairments in reward-guided behavioral shifting are reversed by subsequent amygdala lesions (Stalnaker et al., 2007) and the suggestion that neurotoxic amygdala lesions might even improve ODR performance suggests opposition between the roles of the areas.

**Figure 3:**
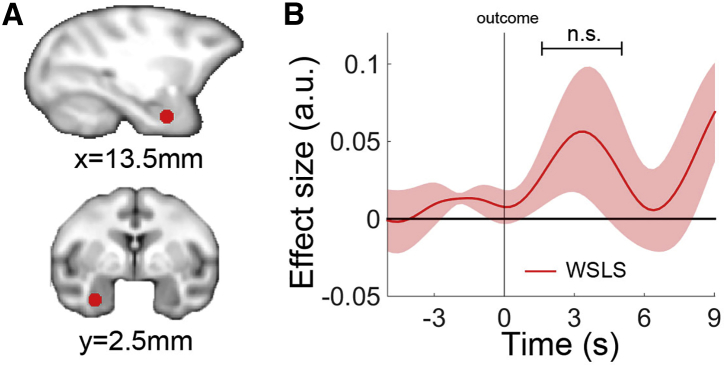
Lose-Shift Signal in the Amygdala (A) The amygdala ROI (14, −3, −13; red) in sagittal view (top panel) and coronal view (bottom panel) for BOLD activity extraction. (B) There was no clear WSLS (green) signal in the amygdala.

We placed an ROI over basolateral and lateral nuclei of the amygdala (14, −3, −13; Figure 3A), the region investigated in previous studies (Morrison et al., 2011; Paton et al., 2006; Saddoris et al., 2005). Three out of four monkeys carried a positive WSLS signal at the outcome phase that was similar to one seen in lOFC, although the group average effect across the four monkeys was not significant (*t*_*3*_ = 1.106, p = 0.350; Figure 3B).

**Figure 4:**
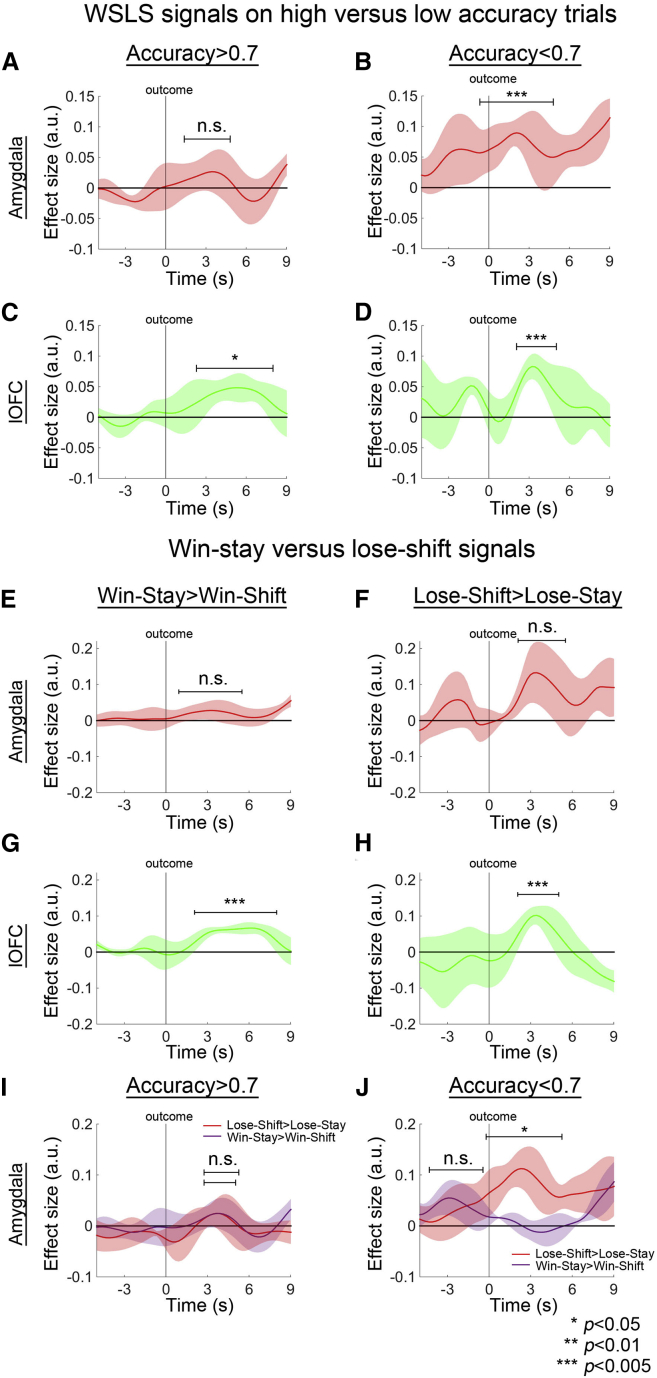
Distinctive Features of lOFC and Amygdala WSLS Signals First, we split the WSLS signals by whether a trial occurred in a period when accuracy was below 0.7. The amygdala did not encode a WSLS signal when monkeys’ accuracies were high (A) but did so when accuracies were low (B). In contrast, the lOFC encoded WSLS signals no matter whether accuracies were high or low (C and D). Second, we split the WSLS signal by whether it was a win or lose trial. In other words, win-stay and lose-shift signals were investigated separately. The amygdala did not have a win-stay signal (E), but three monkeys encoded a positive lose-shift signal (F). In contrast, both win-stay and lose-shift signals were seen in the lOFC (G and H). In the amygdala, the lose-shift signal was strongest, and statistically significant, when focusing on trials with low accuracies (I and J).

Because the amygdala’s role in reward-guided behavioral reversal may be limited to certain stages or events during learning (Rudebeck and Murray, 2008), we analyzed the WSLS signals on trials prior to the onset of the post learning phases (when mean accuracy calculated by using a moving window of five trials was >0.7) and also during the five trials after first reaching the 0.7 accuracy criterion of the post-learning phase, as opposed to trials with accuracies exceeding 0.7. Presumably monkeys were still learning which stimulus was correct on these low-accuracy, but not high-accuracy, trials. Interestingly, we found that the amygdala had a strong WSLS signal when accuracy was low (*t*_3_ = 12.387, p = 0.001; Figure 4B) but not when accuracy was high (*t*_3_ = 0.843, p = 0.461; Figure 4A). The results cannot be a consequence of greater statistical power in the low-accuracy task phases; if anything statistical power was lower in the low-accuracy task phase because fewer trials were assigned to this task phase. In contrast, WSLS signals were present in both the low- (*t*_3_ = 9.292, p = 0.003; Figure 4D) and high-accuracy trials in the lOFC (*t*_3_ = 3.285, p = 0.046; Figure 4C).

Because there were more unrewarded trials in the low-accuracy trials when the amygdala exhibited a WSLS signal, we hypothesized that the amygdala WSLS signal might actually be specific to lose-shift trials. We tested this by analyzing win and lose trials separately. We found that a win-stay signal was absent in the amygdala (*t*_3_ = 1.202, p = 0.315; Figure 4E). Although there appeared to be a lose-shift signal on unrewarded trials it was statistically insignificant (*t*_3_ = 0.980, p = 0.399; Figure 4F). However, when we split the win-stay and lose-shift signals in the amygdala according to accuracy of the task phase, we found a strong lose-shift signal (*t*_3_ = 4.025, p = 0.028; Figure 4J) when accuracy was low, although the effect was not significant on lose-shift trials in high-accuracy task phases (*t*_3_ = 0.909, p = 0.430; Figure 4I). Additional analyses showed that the lose-shift signal was not confounded by reward expectation and reward prediction error (Figures S6A and S6B), although in line with previous studies (Belova et al., 2007) we were able to find an additional effect of reward expectation on amygdala activity. In addition, we found that the lose-shift signal had a spatial property; such a pattern of activity is consistent with evidence that amygdala neurons combine information about reward and space (Peck et al., 2013) (Figures S6C and S6D). By contrast, the lOFC exhibited both win-stay (*t*_3_ = 12.751, p = 0.001; Figure 4G) and lose-shift signals (*t*_3_ = 8.936, p = 0.003; Figure 4H).

**Figure 5:**
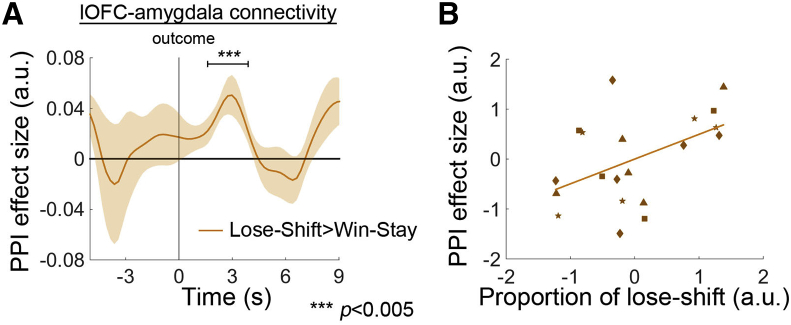
PPI between lOFC and Amygdala that Guided Behavioral Change (A) lOFC and amygdala exhibited stronger connectivity when monkeys performed lose-shift rather than win-stay behavior. (B) Larger PPI effect sizes were related to higher proportions of lose-shift behaviors. Each type of marker symbol in (B) represents data from one animal.

Because the lOFC and amygdala exhibited a similar lose-shift signal but different win-stay signals, it is possible that the connectivity between these two regions could also be modulated as a function of the outcome of a choice. To test this, we used the psychophysiological interaction (PPI) test (Friston et al., 1997) commonly used in fMRI studies. In this PPI analysis, we examined the impact on amygdala activity of the interaction of a physiological parameter, the lOFC activity (as indexed by the lOFC BOLD time series), and a psychological parameter indexed by a task variable (a contrast between the lose-shift contingency and the win-stay contingency). We focused on the interaction of these two influences (PPI effect) but took care to include both main effects in our analysis (O’Reilly et al., 2012). The lOFC and amygdala were more strongly coupled during lose-shift events than during win-stay events (*t*_3_ = 9.215, p = 0.003; Figure 5A). There was a relationship between the effect size of this change in coupling in different sessions and behavioral variation in the sessions; larger sizes of lose-shift coupling between lOFC and amygdala were present in testing sessions in which a higher proportion of lose trials were lose-shift trials (even after controlling for the proportion of win trials that were win-stay trials; Figure 5B; *r* = 0.476, p = 0.040).

### Reinforcement Learning in the Ventral Striatum

The orbital-striatal circuit may also have a role in reinforcement learning. In the ventral striatum, we found a significant WSLS signal (Figures S7A and S7B); however, WSLS had no impact on the functional connectivity between ventral striatum and lOFC (Figure S7C). In other words, our results do not show any evidence that the WSLS signal in OFC was dependent on interactions with ventral striatum. Instead, the connectivity between the lOFC and the ventral striatum is modulated as a function of reward prediction error (Figure S7D). It is possible that the orbital-striatal circuit has a role in updating the value of the options, whereas adaptive behavioral change is driven by the lOFC or OFC in interaction with amygdala. Such arguments are compatible with previous studies that have also suggested that OFC-dopaminergic interactions are important during value updating (Takahashi et al., 2011) and with evidence that there are neurons in anterior but not posterior parts of the striatum in which activity reflects recently updated stimulus values as opposed to the longer-term history of reward associated with a stimulus (Kim and Hikosaka, 2013). While it is clear that such flexible value representations in striatum influence behavior and that they may do so via the D1-dependent direct output pathway of the striatum (Yawata et al., 2012), it is possible that these influences are not always exerted via connections with cortex but perhaps also via other subcortical structures (Yasuda and Hikosaka, 2015).

### Non-contingent Learning in the Amygdala

So far the analyses have been consistent with a view of lOFC and amygdala as interacting in order to bring about behavioral change because the areas share a signal predicting choice shift after a failure to obtain reward. Variation in the size of the amygdala signal and in degree of modulation in lOFC-amygdala coupling predicted lose-shift behavior (Figure 5). However, the view of the lOFC and amygdala as cooperating in the exchange of information to guide learning is at odds with demonstrations that amygdala lesions improved some aspects of ODR performance in macaques and rescued the ODR impairment caused by OFC lesions in rats (Rudebeck and Murray, 2008; Stalnaker et al., 2007).

One way to reconcile views of the amygdala as either helping or hindering reward-guided learning is by considering the possibility that it makes a particular type of contribution to reward-guided learning. In addition to learning precise contingent relationships between predictive stimuli and reward it is clear that both in animals and humans a “spread of reward effect” occurs whereby the reward delivered after one choice “spreads” forward to the next trial so that it also reinforces the choice made on the next trial (Thorndike, 1933; Walton et al., 2010). Such a learning mechanism is unproblematic in many situations if the same choice is repeated trial after trial. It is problematic when a learner is shifting rapidly between choices on consecutive trials because spread of reward effect means that the credit for an outcome on one trial may be partly misassigned to a different choice made on the subsequent trial that may actually have followed rather than preceded the outcome. If the amygdala still encodes information about reward on trial t-1 that had actually been received in response to a choice, potentially a different choice, on trial t then it should be possible to observe an impact of the previous trial’s reward on the amygdala BOLD signal.

**Figure 6:**
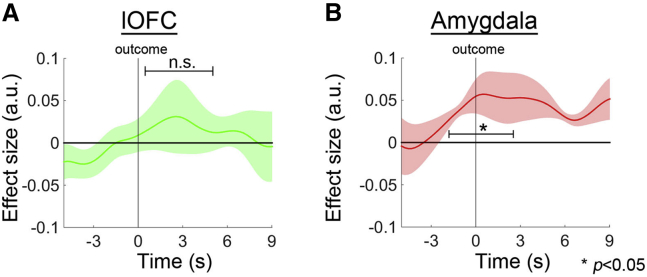
Previous Reward Signals in the lOFC, ACC, and Amygdala (A) The lOFC did not carry signals related to whether or not a reward had been delivered on the previous trial at the time of the outcome phase on the subsequent trial. (B) The amygdala encoded the reward of the previous trial at the time of the decision and outcome phases of the current trial.

**Figure 7:**
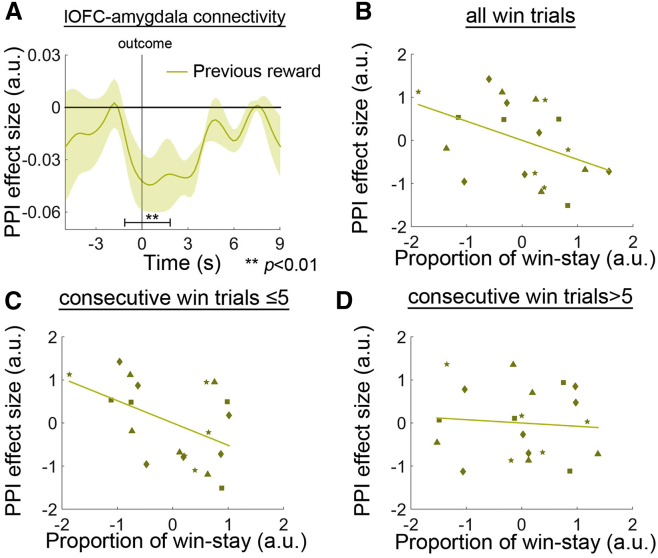
PPI between lOFC and Amygdala that Avoided Irrelevant Reward Information (A) The lOFC-amygdala connectivity was negatively modulated as a function of the previous reward. (B–D) Testing sessions with stronger negative modulation was marginally related to more stay decisions after a win trial when all trials were considered together (B) and statistically significantly related to the presence of more stay decisions when analysis was focused on the first five consecutive win trials (C). There was no relationship between the same neural signal and behavior after five consecutive win trials (D). Each type of marker symbol in (B)–(D) represents data from one animal.

Whether or not reward had been received on a previous trial did not influence decision and outcome-related activity on subsequent trials in lOFC (*t*_*3*_ = 0.849, p = 0.458; Figure 6A). In contrast, the amygdala carried a previous reward signal throughout the course of the subsequent trial’s decision and outcome phases (*t*_*3*_ = 5.552, p = 0.012; Figure 6B), suggesting that the amygdala might mediate the assignment of the reward from a previous trial to an option chosen on the current trial and hence mediate a spread of reward effect. In a PPI analysis in which we examined the interacting influences of lOFC activity and the previous trial’s reward on amygdala activity, we found negative lOFC-amygdala coupling as a function of previous reward delivery (*t*_3_ = −7.207, p = 0.006; Figure 7A). In other words, the lOFC-amygdala connectivity was weaker when the amygdala itself was showing a signal related to reward delivery on the previous trial.

In order to investigate whether the lOFC-amygdala coupling could be simultaneously modulated as a function of a previous reward in a negative manner and as a function of lose-shift behavior in a positive manner, we included both PPI regressors at the same time in one analysis. The results for the two PPI effects remained the same (Figure S8), suggesting that both types of modulation co-existed.

One important consequence of non-contingent learning is that when consecutive choices alternate and only some are rewarded, it is more unlikely that an animal will stay with the rewarded option because credit for the reward may be misassigned to a subsequent incorrect choice. The “credit” for a non-reward may also be misassigned to a subsequent correct choice. Negative lOFC-amygdala coupling appears to reduce such credit assignment problems; in win trials, we found that stronger negative coupling between the lOFC and amygdala, as a function of previous reward, was marginally related to more frequent future win-stay decisions (even after controlling for the proportion of lose trials that were of the lose-shift type; Figure 7B; *r* = −0.425, p = 0.070). When appraising this marginally significant effect, it is important to realize that when a series of win trials are performed consecutively, then any effect of non-contingent learning should be most obvious on the first few consecutive win trials that occurred early in the series. Any disruptive impact of reward spread should diminish after repeatedly choosing the same option correctly many times because even if there was non-contingent learning, the credit for the previous reward could no longer be spread to another choice. We found that the increased lOFC-amygdala negative coupling that was linked to receipt of a previous reward was related to a higher proportion of win-stay choices in the first five consecutive win trials (even after controlling for the proportion of lose trials that were lose-shift trials and the proportion of win trials occurring after five previous consecutive win trials that were win-stay; Figure 7C; *r* = −0.476, p = 0.046). However, the lOFC-amygdala negative coupling was not related to the proportion of win-stay choices made after the same win choice had already been repeated more than five times (even after controlling for the proportion of lose trials that were lose-shift trials and the proportion of win trials occurring on the first five consecutive win trials that were win-stay; Figure 7D; *r* = −0.085, p = 0.738).

### Experiment 2: Probabilistic Learning Task

**Figure 8:**
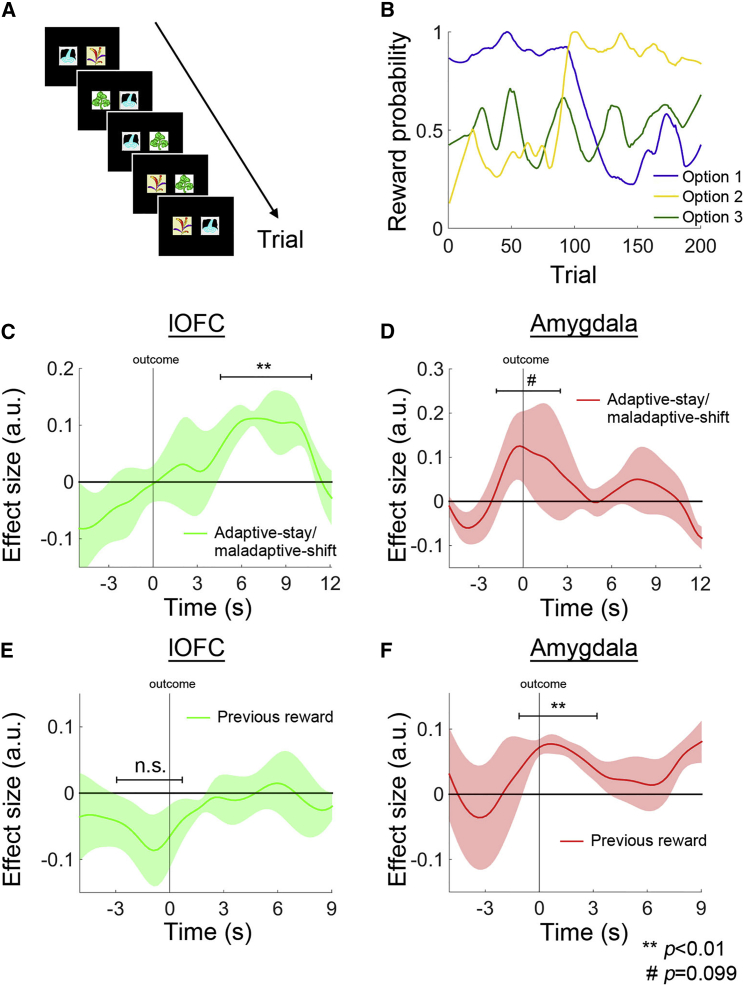
Probabilistic Learning Task: Experiment 2 (A) On every trial, two out of three options were offered to the animals to choose. (B) Each option was associated with a probability of reward, as opposed to being linked in a deterministic manner as in the ODR task in Experiment 1. Instead of relying on the outcome of the previous decision and choosing according to a WSLS strategy, animals had to integrate the reward history of an option over an extended number of trials to make adaptive choices. (C) When the value of the chosen option was larger than that of the unchosen option (adaptive), animals should stay with the same choice when the same pair of options was offered on the next trial; however, animals should shift to the unchosen option when the value of the chosen option was smaller than the unchosen option (maladaptive). In other words, animals should follow an adaptive-stay/maladaptive-shift (ASMS) strategy and use of just such a strategy was associated with lOFC (16, 8, −4) activity. (D) In contrast, the amygdala only showed a marginally significant ASMS signal. (E and F) As in the ODR task in Experiment 1, a signal related to whether or not reward was delivered on the previous trial was absent in the lOFC (E) but present in the amygdala (F).

In the deterministic ODR task in experiment 1, the lOFC signal was related to WSLS behavior, which is an optimal strategy for maximizing reward intake in this task. However, it is unclear whether lOFC was strictly related to WSLS behavior only or whether it also had a role in driving other kinds of adaptive behavior when animals encountered a different kind of task. Next, we trained monkeys to perform a probabilistic learning task where there were three options in a day’s testing session. However, on each trial, only two out of the three options were offered for the monkey to choose between (Figure 8A). The options were each associated with a probabilistic, rather than deterministic, reward and the reward probabilities drifted over the course of the testing session (Figure 8B). To behave adaptively in this task, animals should not employ the same identical WSLS strategy as previously because an option that was frequently associated with a reward could still on occasion yield no reward when chosen and equally a poor option could still be rewarded occasionally. Instead monkeys should now adapt the WSLS to take into account not just whether the last outcome was a reward or error, but also the average recent rates of reward associated with the option just chosen and the alternative option available. To perform adaptively in this task, when the value of the chosen option was larger than that of the unchosen option (“adaptive”), animals should “stay” with the same choice; however, animals should “shift” to the unchosen option when the value of the chosen option was smaller (recent choices have been “maladaptive”). In other words, animals should follow an adaptive-stay/maladaptive-shift (ASMS) strategy in this task.

We focused on trials when the same pair of options was offered on two consecutive trials—those were the trials when the ASMS strategy was particularly important. We found, in experiment 2, that this adaptive behavior was related to the signal in the same lOFC region (16, 8, −4; *t*_3_ = 7.3964, p = 0.005; Figure 8C) in which we had found the WSLS signal for guiding adaptive behavior in the ODR task in experiment 1. In experiment 2, we again found a non-contingent learning or spread of reward signal in the amygdala (14, −3, −13); amygdala activity reflected whether reward had been delivered on the previous trial (*t*_3_ = 12.258, p = 0.001; Figure 8F) and there was no evidence for a previous reward signal in the lOFC (*t*_3_ = −0.729, p = 0.519; Figure 8E). Finally, again in experiment 2, we replicated the finding that signals relating to adaptive behavioral change were weak in the amygdala; we only found a marginally significant ASMS signal in the amygdala (*t*_3_ = 2.362, p = 0.099; Figure 8D). It is intriguing that this signal, despite not reaching significance, began to evolve prior to the onset of the outcome event. This may reflect the fact that in experiment 2 ASMS behavior was not contingent just on the last outcome but on whether or not each option had, on average, been associated with reward over the last few trials (note that the ASMS regressor reflected past outcome history over several trials not just the last one). The weak ASMS signal that does exist in the amygdala therefore appears to be related to the previous reward signal that we had also found in the amygdala in both experiments (Figure 6B).

Finally, we performed multilevel modeling to compare the strength of WSLS/ASMS signal and previous reward signal (signal type) across lOFC and amygdala (brain region) using data from both experiments. There was no significant main effect of brain region (*F*_1,10.5_ = 0.087, p = 0.774). The WSLS/ASMS signal was significantly larger than the previous reward signal (*F*_1,10.5_ = 12.912, p = 0.005). Importantly, there was a significant signal type by brain region interaction effect (*F*_1,3.18_ = 19.909, p = 0.018). A post hoc analysis showed that WSLS/ASMS signal was significantly stronger in lOFC than in amygdala (*F*_1,19.9_ = 9.377, p = 0.006), whereas previous reward signal was significantly stronger in amygdala than in lOFC (*F*_1,11.8_ = 12.713, p = 0.004).

## Discussion

The amygdala and OFC have been linked to the flexible use of reward information to guide behavior. However, the nature of the link has recently become controversial. For many years, flexible reward-guided behavior has been investigated using the ODR task in which animals have to learn first that one stimulus is associated with reward but that subsequently only an alternative stimulus is associated with reward. The identification of amygdala and OFC with ODR reflects a long history of demonstrations that lesions impair ODR in macaques (Butter, 1969; Izquierdo et al., 2004; Jones and Mishkin, 1972; Rudebeck and Murray, 2008) and more recent demonstrations that the responses of individual neurons in macaque OFC and amygdala track changes in the reward associations of stimuli (Morrison et al., 2011; Paton et al., 2006; Rolls, 2000).

Unfortunately, this attractively coherent picture of OFC and amygdala function has been called into question by recent demonstrations that OFC and amygdala lesions in macaques do not disrupt ODR when the lesions are made by neurotoxin injection (Izquierdo and Murray, 2007; Kazama and Bachevalier, 2009; Rudebeck et al., 2013b). Confusingly, neurotoxic lesions made in the OFC of new world monkeys, such as marmosets (Dias et al., 1996; Rygula et al., 2010), and of rodents (Saddoris et al., 2005; Schoenbaum et al., 2003, 2007) impair ODR. The degree of homology between the OFC in old world primates such as macaques and humans on the one hand and rodents and new world primates such as marmosets has been questioned (Passingham and Wise, 2012). Nevertheless, in both rodents and macaques there is now evidence that selective lesions of the amygdala lead to improvements, not impairments, in ODR performance (Izquierdo et al., 2013; Rudebeck and Murray, 2008; Stalnaker et al., 2007).

By training macaques to perform ODR in the MRI scanner, we attempted to obtain a new perspective into the neural basis of flexible reward-guided behavior in primates. We found an lOFC region (Figures 2B and 2C) that showed a particularly strong WSLS signal as monkeys used the current outcome of a choice to adaptively guide future behavior (Figure 2G). The effect cannot be related to a verbal mediation strategy dependent on adjacent ventral frontal brain regions associated with language because the lOFC data come from macaques that are non-linguistic. Stronger signals in this region were related to testing sessions with higher learning rates (Figures 2I and S7). Moreover, by training the same animals to perform in a probabilistic learning task in experiment 2, we found that the same lOFC region encoded a signal that was related to an ASMS behavior when this became the best strategy (Figure 8C). Hence, we argue that this lOFC region is not constrained to carry a WSLS signal, but rather it has a role in directing behavior that is adaptive to the context, given the recent distribution of reward to choices, in order to maximize future reward.

The location of the lOFC region we identified, which extends from anterior insula into the orbital part of area 12/47, has important implications for understanding some of the past controversy and reconciling apparently conflicting patterns of results. First, area 12o, which is in or near this region, has a unique connectivity profile that interconnects medial and lateral regions on the orbitofrontal surface (Carmichael and Price, 1995b; Kondo et al., 2005). Second, the region lies just lateral to the central OFC region (areas 11 and 13) where the effects of neurotoxic lesions were studied (Kazama and Bachevalier, 2009; Rudebeck et al., 2013b). It would, however, be likely to have been partially deafferented by aspiration lesions in the central OFC region; connections running to and from this region in the amygdalofugal pathway and uncinate fascicle are immediately subjacent to the central OFC (Croxson et al., 2005; Jbabdi et al., 2013). Although the amygdala’s connections to medial OFC are often emphasized, the medial OFC is only one of four regions with especially high levels of interconnection with amygdala (Amaral et al., 1992). The posterior lOFC region we identified here is one of the other regions with strong amygdala connections (Amaral et al., 1992; Carmichael and Price, 1995a; Ghashghaei and Barbas, 2001). In addition, anatomical connections with inferior temporal and perirhinal cortex areas concerned with higher-order visual pattern processing are also prominent in lOFC (Carmichael and Price, 1995b; Kondo et al., 2005). Such connections suggest that this part of the OFC may have an important role to play in associating stimuli with reward in tasks such as ODR. Further, because BOLD reflects synaptic activity within cortex and not just the spiking output of cortex (Logothetis et al., 2001), our fMRI experiment may have been particularly sensitive to brain regions in which synaptic input was task related. Similarly, if it is true that lOFC-amygdala interactions are important for good task performance, then one would expect that lesions that directly affect the parts of OFC with amygdala inputs and outputs will be the most disruptive ones. This, of course, does not preclude the existence of task-related spiking activity in adjacent lOFC regions (Morrison et al., 2011; Rolls, 2000). In summary, lesion and neuroimaging approaches may both emphasize the brain regions where there are input and output connections that mediate the interactions with other areas during a cognitive process. Therefore, we do not argue that the lOFC is the only OFC region important for flexible reward-guided behavior. Our contention is, however, that the lOFC has a special importance that may explain why lesions that do not include it or deafferent it do not cause impairments. Indeed, in an early study by Butter (1969), poor object reversal learning was not seen in monkeys with posteromedial OFC or anterior OFC lesions, but only observed in monkeys with total OFC or lOFC lesions (including the same region we identify here). The different approaches for examining the role of OFC—lesion, neuroimaging, and neurophysiological recording—each have different strengths and biases but converge in suggesting a picture of how interactions between neural activity distributed in this area and in interconnected areas such as amygdala and ventral striatum are causally important for reward-guided learning. The human homolog of the same lOFC region (Neubert et al., 2015) was also found to be active in human fMRI ODR experiments (Ghahremani et al., 2010; O’Doherty et al., 2001).

We found that the amygdala also carried task-relevant signals that guided adaptive behavioral change. These signals, however, were only prominent at early stages of learning and when the monkeys failed to obtain a reward (Figures 4I and 4J). Moreover, we were able to show that activity in the lOFC and amygdala was more strongly coupled as a function of a lose-shift signal observed when animals were going to shift to an alternative option after failing to obtain reward (Figure 5). Those signals are likely to be associated with the basolateral nucleus of the amygdala, which is particularly interconnected with posterior lOFC (Ghashghaei and Barbas, 2001). Although the relatively poor temporal resolution means that BOLD coupling analyses are unable to infer the direction of signal propagation, it is likely that the lose-shift signal in the lOFC originates, at least in part, from the amygdala. Morrison and colleagues (2011) recorded both OFC and amygdala neuronal activity with the high temporal precision that electrophysiological recording techniques afford and showed the local field potentials, which are thought to be similar to BOLD signal, in amygdala exerted a stronger influence on OFC activity, as opposed to an OFC-to-amygdala influence, when option value updating was required. After learning, they showed that the latency of the activity of individual OFC neurons in response to an expected reward was faster than the amygdala. This highlights the importance of the dynamic change in connectivity between OFC and amygdala in guiding behavior both during and after reversals.

Our experimental design also allowed us to understand why amygdala lesions might, in some cases, improve ODR performance (Rudebeck and Murray, 2008; Stalnaker et al., 2007). This is because we also found a second type of signal in the amygdala that might be disruptive to task performance and that might be suppressed by the OFC. It is becoming clear that in a number of brain areas reward memories are represented over multiple timescales—a short timescale that contains information about the most recent reward only and over long timescales that integrate over both the recent and remote histories of reward events (Bernacchia et al., 2011; M. Wittmann, B.K.H.C., M.F.S.R., and colleagues, unpublished data). Multiple timescale reward information can be useful for assessing whether, on average, the environment is getting better or worse, but it can be problematic if long timescale reward memories interfere with the process whereby specific reward events are associated with specific preceding events. Under such circumstances learning is slowed down by a “spread of reward” effect (Thorndike, 1933), such that the credit for a previous reward delivered after a previous choice is mistakenly assigned to the current choice. We showed that the amygdala contained a signal that encoded the outcome of the previous trial even though this was no longer relevant to assessing the correctness of the current trial’s response in both experiments 1 and 2 (Figures 6B and 8F). The lOFC did not carry a similar signal (Figures 6A and 8E) and in fact coupling between lOFC and amygdala became weaker when the amygdala carried a signal that was related to the irrelevant previous outcome (Figure 7A). Note that the negative lOFC-amygdala coupling that occurs as a function of prior reward is of the opposite sign to the positive lOFC-amygdala coupling that occurs as a function of lose-shift signals. Because both patterns of lOFC-amygdala coupling are not simply correlations in overall activity levels, but instead arise in relation to particular event-related signals, they can occur simultaneously. This suggests that the lOFC could also have a role in selecting reward memories in a relevant timescale (i.e., memory that only involved the immediate reward in deterministic ODR in experiment 1) at the expense of other irrelevant timescales (i.e., memories that involved the outcome of the previous trial in the deterministic ODR in experiment 1). Consistent with this interpretation, we found that more negative modulation in lOFC-amygdala coupling, as a function of the previous reward, was related to more win-stay behavior (Figures 7B and 7C). This supports the view that the lOFC could interact with other brain regions to select relevant information and reject irrelevant information to generate adaptive behaviors (Chau et al., 2014). If this view is correct, then it would also predict that lOFC lesions would lead animals to be more likely to make fallacious links between a current choice and a previous reward than control animals and indeed this is exactly what has been observed (Noonan et al., 2010; Walton et al., 2010).

Nevertheless, it remains unclear how the spread of reward effect is best conceived as operating during reinforcement learning. One possibility is that a reward obtained in the past would have an impact on the slow-drifting emotional state of an individual that partially shifts the perception of the valence of an outcome that follows the current choice. Future studies focusing on non-contingent learning would be useful for further understanding its mechanism of operation.

In summary, we show that lOFC and amygdala both carry signals related to reward-guided behavioral change but that there are fundamental differences in the nature of the signals carried by each area. LOFC activity is consistently related to the use of WSLS or ASMS rules that facilitate good task performance but in some other cases the signals are detrimental to good deterministic ODR performance. For example, an aspect of amygdala activity is related to previous reward outcomes that are irrelevant for assessing the success of the current choice. Variations in the strengths of signals in each area and in inter-areal signal coupling are associated with variations in different aspects of performance. The patterns of activity that we found suggest two new predictions. First, excitotoxic lesions that include lOFC should impair ODR. Second, excitotoxic amygdala lesions might impair performance on tasks in which it is necessary to integrate information about reward received over several trials and not just on the most recent trial as in deterministic ODR.

## Experimental Procedures

### Subjects

Four male rhesus monkeys (*Macaca mulatta*) were involved in the experiment. They weighed 10.4–11.9 kg and were 7 years of age. They were group housed and kept on a 12 hr light dark cycle, with access to water 12–16 hr on testing days and with free water access on non-testing days. All procedures were conducted under licenses from the United Kingdom (UK) Home Office in accordance with the UK The Animals (Scientific Procedures) Act 1986. One testing session from one animal was excluded due to excessive motion during scanning. Details for the behavioral training are described in Supplemental Experimental Procedures.

### Experimental Task

In our deterministic ODR task (experiment 1), subjects needed to choose repeatedly between two stimuli that were novel in each testing session (Figure 1A). Each trial began with a blank screen (inter-trial interval; 5–7 s). Two stimuli were presented on the left and right sides (stimuli positions were randomized on every trial) on the screen, and subjects had to choose an option by touching one of two infra-red sensors placed in front of their left and right hands that corresponded to the stimuli on the screen (decision phase, mean RT = 1,089 ms after excluding trials with RT > 10 s). If the correct option was chosen, the unchosen option disappeared and the chosen option remained on the screen and a juice reward was delivered. If the incorrect option was chosen, both stimuli disappeared and no juice was delivered (outcome phase; 1.5 s). Each reward was composed of two 0.6 ml drops of blackcurrant juice delivered by a spout placed near the subject’s mouth during testing. A given session ended when subjects performed 150 rewarded trials (on average 183.4 trials in total). The task used a deterministic reversal schedule such that each session began with one stimulus that always led to a reward and another stimulus that always led to no reward. The stimulus-reward contingencies reversed for the first time after 50 rewarded trials had been performed and then again after a further 50 rewarded trials were performed. No cue signaled the change in stimulus-reward assignment (Figure 1B). Each animal performed four to six sessions in the MRI scanner.

As in the deterministic ODR task, in the probabilistic learning task (experiment 2), subjects chose between two stimuli on each trial. Instead of having the same pair of stimuli on every trial, two out of three stimuli were randomly drawn for the animals to choose from (Figure 8A). Each stimulus was associated with a reward probability that changed throughout a session (Figure 8B), as opposed to the deterministic reversal design in the ODR task. Each animal performed five to seven sessions in the MRI scanner.

### Behavioral Analysis

Mean accuracies were calculated across subjects within the whole group (Figure 1C), across sessions within subject (Figure 1D), and across trials within session (Figures 1E and 1F). In Figure 1C, trials with the same trial number in a block were averaged across every testing session from all subjects. In Figure 1D, trials with the same block trial number were averaged only across testing sessions from the same subject. In the within-session level (Figures 1E and 1F and subsequent fMRI analyzes), accuracy of each trial was calculated by an averaging window that included the correctness of the current trial as well as that of the next four trials.

### Imaging Data Acquisition

Imaging data were collected using a 3T MRI scanner and a four-channel phased-array receive coil in conjunction with a radial transmission coil (Windmiller Kolster Scientific). fMRI images and reference images for artifact corrections were collected while awake animals were head-fixed in a sphinx position in an MRI-compatible chair. fMRI data were acquired using a gradient-echo T2^∗^ echo planar imaging (EPI) sequence with 1.5 × 1.5 × 1.5 mm^3^ resolution, TR = 2.28 s, TE = 30 ms, flip angle = 90°. Proton-density-weighted images using a gradient-refocused echo (GRE) sequence (TR = 10 ms, TE = 2.52 ms, flip angle = 25°) were acquired as reference for body motion artifact correction. T1-weighted MP-RAGE images (0.5 × 0.5 × 0.5 mm^3^ resolution, TR = 2,500 ms, TE = 4.01 ms) were acquired in separate anesthetized scanning sessions (for details, see Sallet et al., 2013). Preprocessing steps for fMRI data are described in Supplemental Experimental Procedures.

### fMRI Data Analysis

Whole-brain analysis was conducted using a univariate GLM approach with FMRIB’s Software Library (FSL; Smith et al., 2004). We searched for brain regions that encoded future win-stay/lose-shift (WSLS) strategy: maintenance of the same choice on the next trial (“stay”) after a reward outcome on the current trial (“win”) and shifting to the alternative choice (“shift”) after a no-reward (“lose”) outcome on the current trial. To do this, we applied a GLM to every testing session that included the following regressors: a binary regressor describing whether the monkey followed win-stay/lose-shift or win-shift/lose-stay (WSLS regressor) on the next trial, time locked to the onset of the outcome phase on the current trial; a binary regressor (shift) describing whether the monkey stayed with the same option or shifted to the alternative on the next trial, also time-locked to the onset of the outcome phase on the current trial; a binary regressor (reward) indicating whether or not the monkey received any reward on the current trial, time locked to the onset of the outcome period; and a binary regressor (side) describing whether the monkey made a left side or a right side response, time-locked to 0.5 s prior to the onset of the outcome. Note that the WSLS effect can be considered as the interaction term between the main effects of reward and shift regressors. The inclusion of the side regressor should capture variance and noise in the BOLD signal unrelated to the stimulus-based choices that the monkeys were making but purely related to which hand the animal used.

Analyses were first conducted at the individual subject level. Average effects of the GLM across sessions within the same subject were calculated using a fixed-effects analysis. At the group level, analyses were performed using FMRIB’s local analysis of mixed (as opposed to fixed) effects stage 1 and 2 (FLAME1+2) (Beckmann et al., 2003; Woolrich et al., 2004) and using one of the most commonly used and standard cluster-based thresholding criteria of *z* > 2.3 and p < 0.05 cluster-corrected (Worsley et al., 1992) as is now standardly employed in most human neuroimaging studies.

We also conducted analyses on a priori defined ROIs by extracting the BOLD time course from two-voxel radius spherical masks placed over the lOFC (16, 8, −4 in CARET macaque F99 coordinates), central OFC (8, 17, 7), and amygdala (14, −3, −13) signals. Using similar procedures to those used in human fMRI studies (Chau et al., 2014). The mean and standard error (denoted in the figures by lines and shadings respectively) of all the within-subject β weights were calculated across subjects for plotting the effect size time courses. In Figures 2G and 3B, the GLM included regressors describing WSLS (where the shift/stay response occurred on the next trial), shift, and reward as a control regressor (not shown in the figure). In Figure 4, the GLM included only the WSLS regressor. In Figure 6, the GLM included a binary regressor describing whether the outcome on the previous trial had been a reward or not (previous reward) and also WSLS and switch as a control regressors (not shown in the figure). In Figures 8C and 8D, the GLM included regressors describing value difference between the two options, shift, the interaction term between value difference and shift (that is the ASMS regressor), and reward. In Figures 8E and 8F, the GLM included previous reward, value difference, shift, and reward.

Functional connectivity analyses were performed between the lOFC and amygdala and the results of these analyses are shown in Figures 5 and 7. In these analyses, the BOLD time course of the lOFC was used as the physiological regressor to predict the amygdala BOLD signal. In Figure 5, the psychological regressors were shift, reward, and a regressor contrasting between the lose-shift and win-stay components of the WSLS strategy. The psychophysiological interaction (PPI) regressor was computed by taking the product between the lOFC time course and the lose-shift versus win-stay contrast. In Figure 7, the psychological regressors were WSLS, reward, and previous reward. The PPI term was the product of the lOFC time course and the previous reward contrast.

## Author Contributions

B.K.H.C., J.S., and G.P. collected the data. B.K.H.C., G.P., M.P.N., and J.S. trained animals. B.K.H.C., J.S., M.E.W., and M.F.S.R. designed the experiment. J.S. and A.H.B. carried out surgeries. B.K.H.C. and M.F.S.R. analyzed the data. All authors contributed to preparation of the manuscript.

## Supplemental Information

Document S1. Supplemental Experimental Procedures, Figures S1–S8, and Table S1

Document S2. Article plus Supplemental Information
