# Supplementary material for: Contrasting Roles for Orbitofrontal Cortex and Amygdala in Credit Assignment and Learning in Macaques
Source: Neuron. 2015 Sep 2;87(5):1106–18. doi: 10.1016/j.neuron.2015.08.018 (PMC4562909; doi:10.1016/j.neuron.2015.08.018)
Supplement: Document S1. Supplemental Experimental Procedures, Figures S1–S8, and Table S1 [file mmc1.pdf]

Neuron

Supplemental Information

Contrasting Roles for Orbitofrontal Cortex and Amygdala in Credit Assignment and Learning in Macaques

Bolton K.H. Chau, Jérôme Sallet, Georgios K. Papageorgiou, MaryAnn P. Noonan, Andrew H. Bell, Mark E. Walton, and Matthew F.S. Rushworth

Supplemental Information

Contrasting roles for orbitofrontal cortex and amygdala in credit assignment and learning in macaques.

Bolton KH Chau, Jerome Sallet, Georgios K Papageorgiou, MaryAnn P Noonan, Andrew H Bell, Mark E Walton, Matthew FS Rushworth

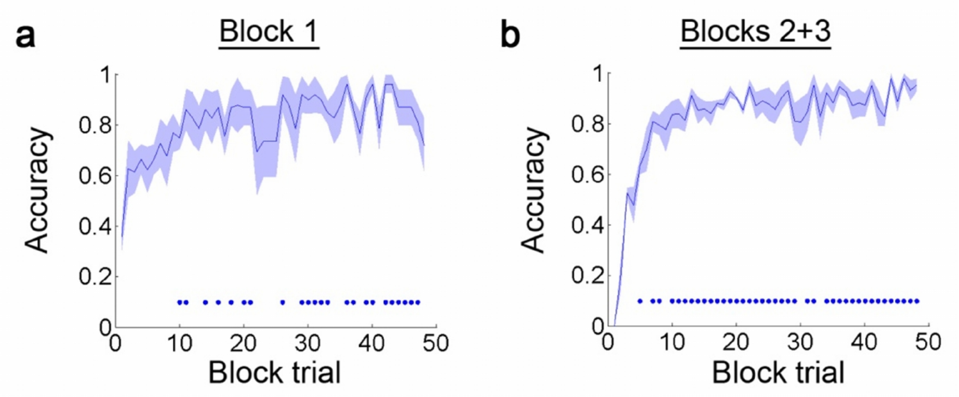

Figure S1 (related to Figure 1). Monkeys showed similar performances in **(a)** block 1, which was the initial discrimination block, and **(b)** blocks 2+3, which were the blocks after the first and second reversals respectively. The raster plots in (a,b) indicate trials in a block with accuracies significantly higher than 0.5 (p<0.05). In both cases monkeys performed poorly on early trials in a block and the accuracies gradually improved. The first few trials in blocks 2+3 were even more inaccurate than those in block 1 because in blocks 2+3 the monkeys encountered a reversal without any prior warning.

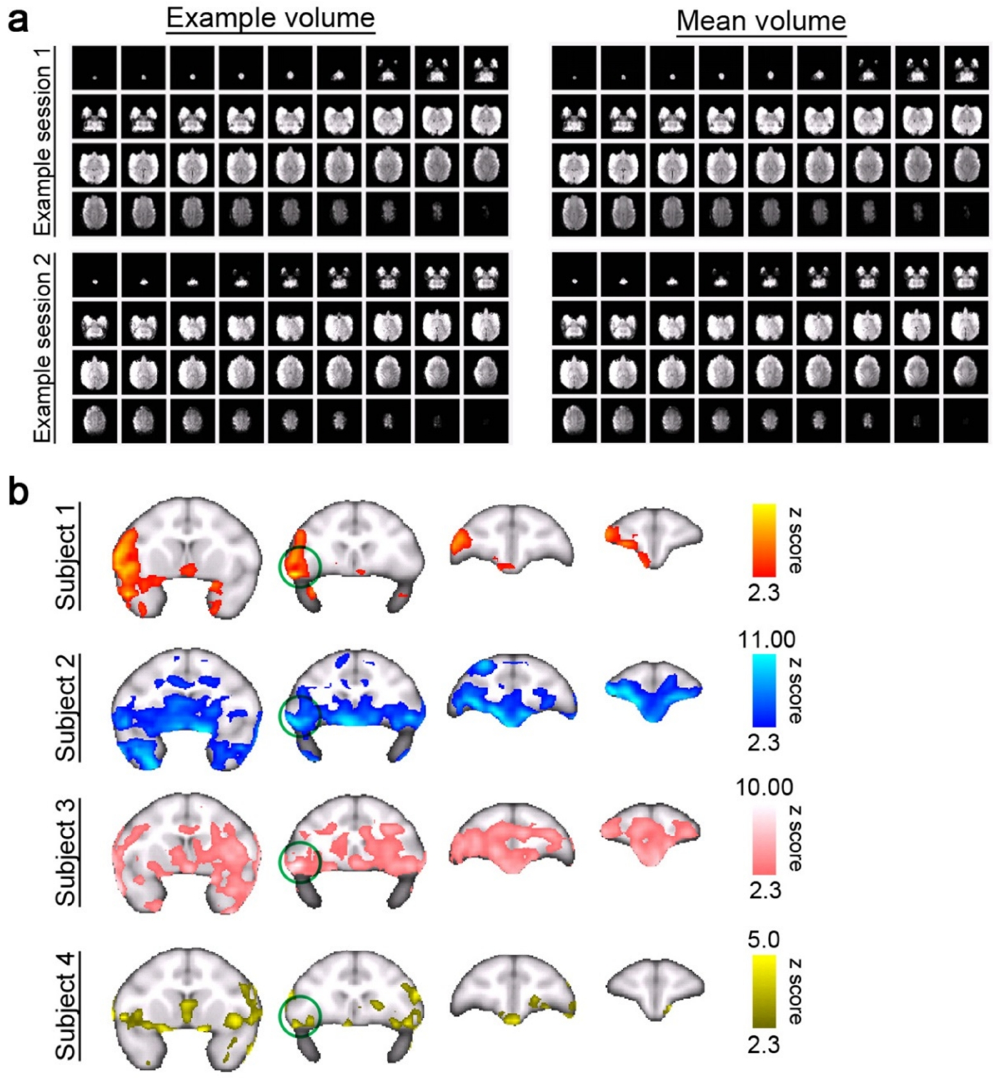

Figure S2 (related to Figure 2). (a) Example functional data and mean functional data from two different animals. **(b)** A signal related to the presentation of the choice outcome was found consistently across subjects in the lOFC (green circle).

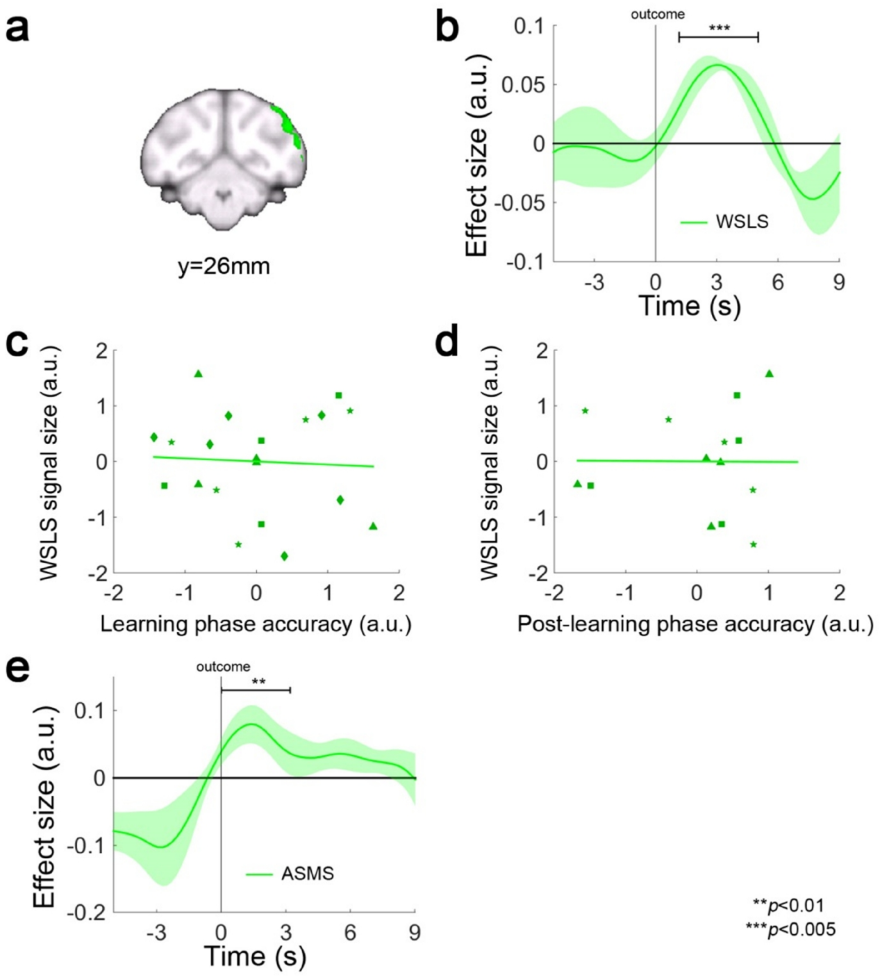

Figure S3 (related to Figure 2). (a) In addition to the lOFC, there was a significant WSLS effect in a posterior cortical region that extended over dorsal V4 and the caudal inferior parietal lobule area 7a. **(b)** Time-course of the WSLS effect (t_3=34.142, p<0.001) in dorsal V4/area 7a. Although the activity in this brain region resembled that in lOFC it appeared less directly related to performance; unlike lOFC, individual and daily variation in activity in this region was not related to individual and daily variation in **(c)** learning phase accuracies (r=-0.058, p=0.816) and **(d)** post-learning phase accuracies (r=-0.008, p=0.973). **(e)** Time-course of ASMS effects (t_3=7.018, p=0.006) in experiment 2 (related to Figure 8). It is possible that activity changes in tandem with the WSLS because attention switch and maintenance effects should be concurrent with the use of the WSLS strategy in experiment 1 and the ASMS strategy in experiment 2.

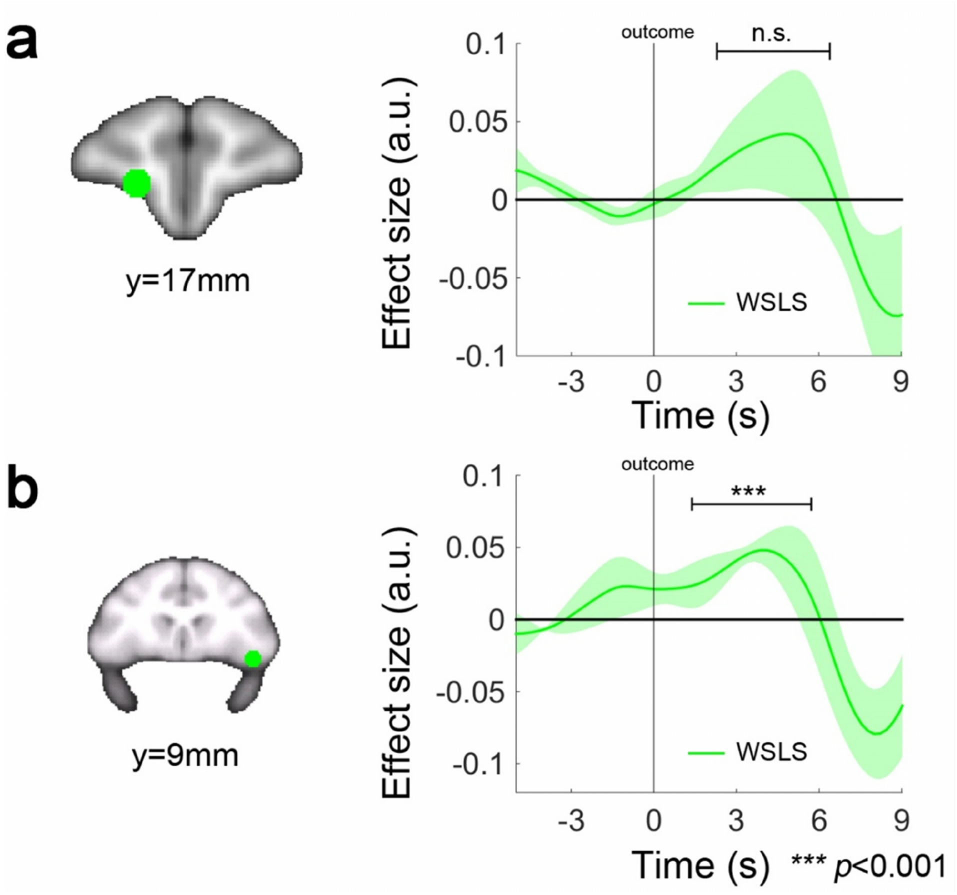

Figure S4 (related to Figure 2). WSLS effects in the central OFC and the left lOFC. **(a)** The central OFC (8, 17, 7; green; left panel) BOLD activity was extracted for ROI analysis. The WSLS signal was not significant in the central OFC (t_3=0.503, p=0.650; right panel). **(b)** The BOLD activity of the lOFC contralateral to that in Fig.2 was extracted for ROI analysis (-19, 9, -3; green; left panel). The WSLS signal was significant in the left lOFC (t_3=15.915, p=0.001; right panel).

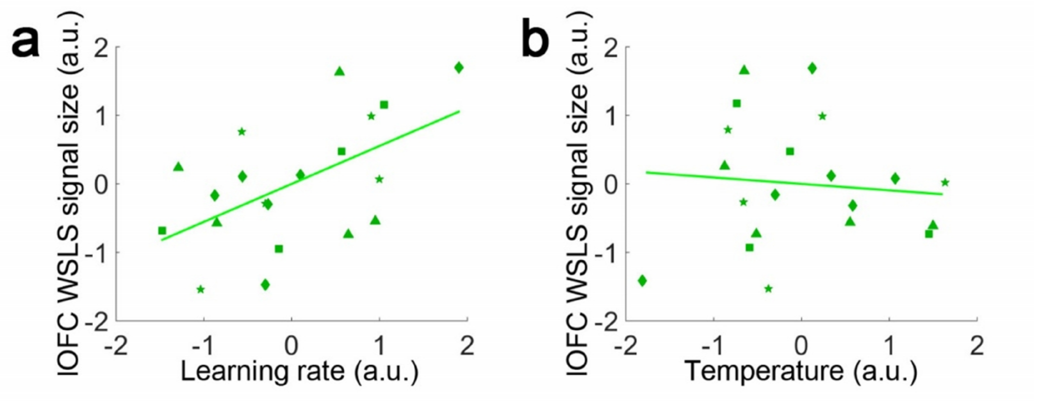

Figure S5 (related to Figure 2). (a) Testing sessions with larger WSLS signals in the lOFC were related to higher learning rates (r=0.551, p=0.015). **(b)** The sizes of the WSLS signal had no relationship with the temperatures, which are indices of choice stochasticity (r=-0.111, p=0.651). The correlation between learning rate and WSLS signal was apparent even after controlling for the effect of temperature. The lack of correlation between the WSLS signal and temperature remained the same regardless of whether or not partial correlation was used to control for learning rate. Each type of marker symbol represents data from testing sessions of one animal.

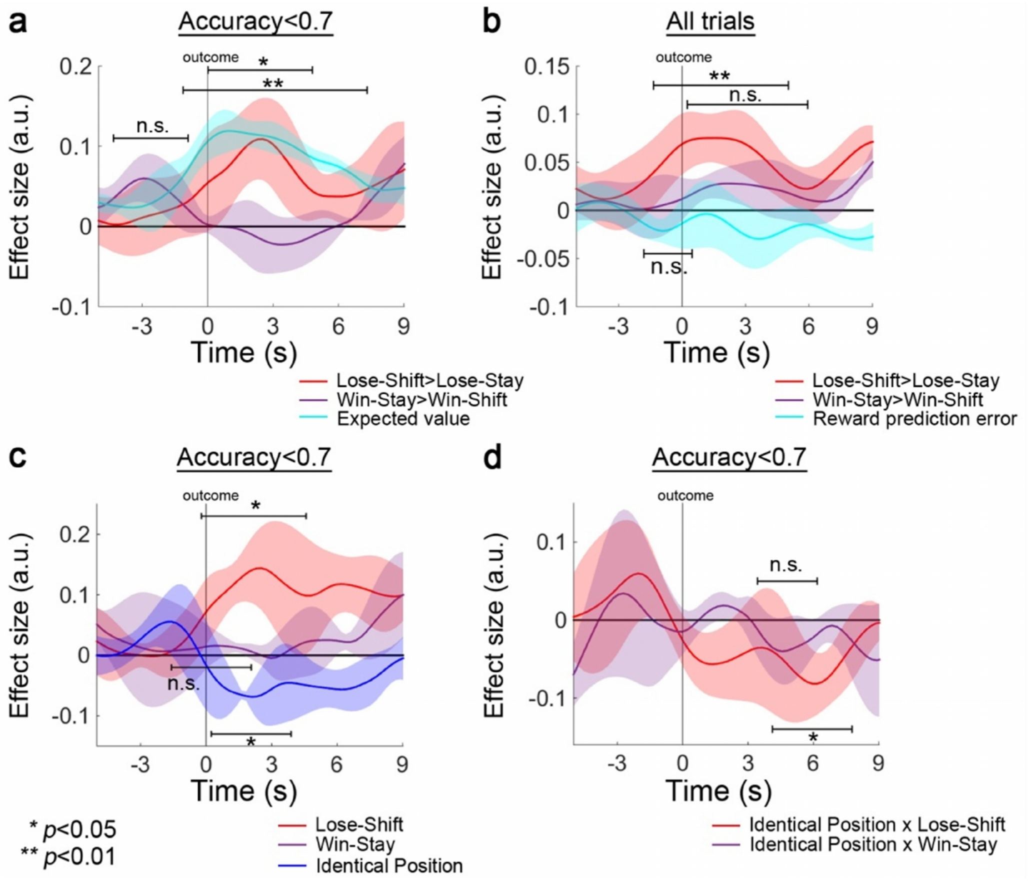

Figure S6 (related to Figure 3). The lose-shift signal in the amygdala was not confounded and contained spatial information. **(a)** Consistent with Figure 4j, on trials when the accuracy was lower than 0.7 there was a strong lose-shift signal (t_3=5.515, p=0.012) and no win-stay signal (t_3=1.316, p=0.280) even when the expected value of the chosen option was entered into the general linear model. However, in addition to the lose-shift signal, we found, as previously reported (Belova et al., 2007), that amygdala activity was modulated as a function of the expected reward value of the option being chosen (t_3=13.740, p<0.001). **(b)** We also used an alternative approach to examine similar issues by including reward prediction error in the general linear model to analyze amygdala activity on all trials. Again, there was a significant lose-shift signal (t_3=6.782, p=0.007) and no win-stay signal (t_3=1.063, p=0.366). Contrary to previous electrophysiology studies (Belova et al., 2007), we did not find a significant reward prediction error signal (t_3=-0.675, p=0.548). This could be due to the fact

that positive and negative prediction error neurons are intermingled in the amygdala and it is difficult to detect the signal using fMRI. Previous studies have shown that reinforcement learning signals in amygdala neurons contains information about the spatial location of the option being chosen (Ousdal et al., 2014; Peck et al., 2013). We investigated whether a spatial code could be found in the amygdala using fMRI. Since the neurons reacting to options in different spatial locations are intermingled, it is not possible to identify space-related signals by simply comparing fMRI activity in the amygdala when an option is positioned on the left and on the right. However, the question can be addressed by repetition suppression – when an identical neuronal response is repeated close in time then the fMRI signal is weaker (Grill-Spector et al., 2006). In addition to the regressors we used in Figure 4i, we added a regressor that describes whether the positions of the two options are identical to those that they occupied on the previous trial. We also included its interaction terms with the lose-shift and win-stay regressors to investigate whether the lose-shift signal carries spatial information. **(c)** Similar to Figure 4i, we found a significant lose-shift signal (t_3=5.621, p=0.011; red) and no win-stay signal (t_3=0.713, p=0.435; purple) in the amygdala. There was a negative effect of identical option position (t_3=-5.087, p=0.018; blue), suggesting that the amygdala contained information about the spatial position of options. **(d)** We found a significant repetition suppression effect in the lose-shift signal (t_3=-9.294, p=0.003; red). In other words the lose-shift signal became weaker when the options were presented in the same position, suggesting that the lose-shift signal in the amygdala also contains spatial information. No repetition suppression effect was found in the "win-stay signal" (t_3=-0.936, p=0.418; purple).

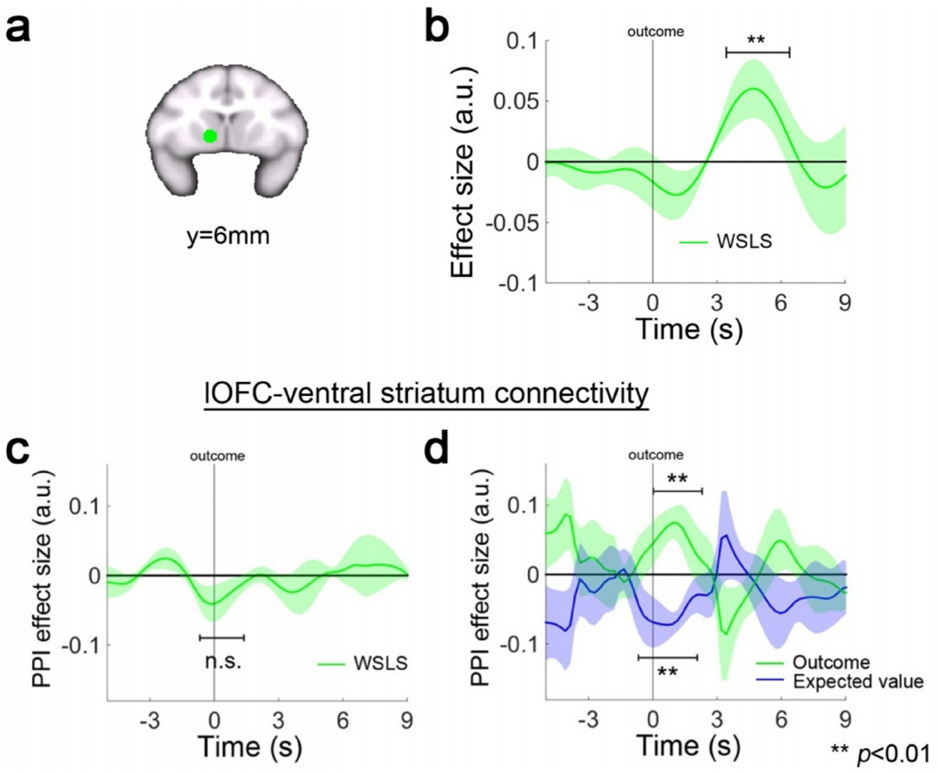

Figure S7 (related to Figure 5). The connectivity between lOFC and ventral striatum was modulated positively as a function of reward prediction error but not modulated by WSLS. **(a)** The ventral striatum (6, 6, 1; green) BOLD activity was extracted for ROI analysis. **(b)** There was a significant WSLS signal in the ventral striatum (t_3=6.274, p=0.008; green). **(c)** However, there was no significant effect of WSLS on the coupling between lOFC and ventral striatum. Reward prediction error is often defined as the difference between the reward outcome and the expected value of a chosen option (Sutton and Barto, 1998). **(d)** There was a simultaneous positive effect of outcome (t_3=7.767, p=0.004) and a negative effect of expected value of the chosen option (t_3=-6,340, p=0.008) on the connectivity between lOFC and ventral striatum. In other words, there is a positive effect of reward prediction error on lOFC-ventral striatum connectivity.

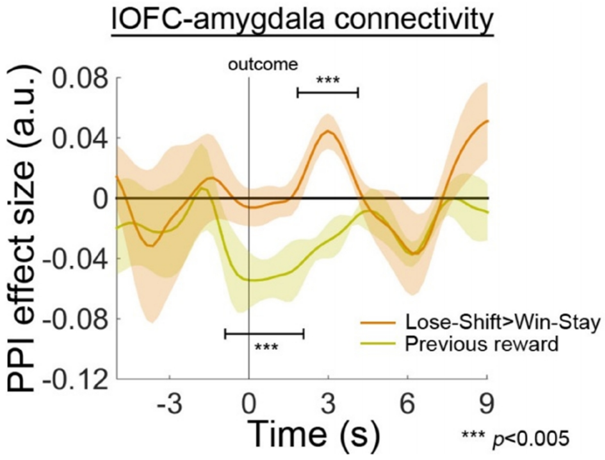

Figure S8 (related to Figure 7)**.** Two types of activity coupling between lOFC and amygdala. When the models in Figures 5a and 7a were combined, similar patterns of PPI were still observed suggesting that the two types of lOFC and amygdala interaction, as a function of lose-shift contingencies and as a function of previous rewards, occurred concurrently. There was a stronger lOFC-amygdala coupling when performing lose-shift behavior than win-stay behavior (t_3=8.857, p=0.003; orange). At the same time, there was a negative effect on the lOFC-amygdala coupling as a function of whether a reward was delivered in the previous trial (t_3=-9.847, p=0.002; yellow).

Table S1. Regions that the activity was modulated by the occurrence of the outcome event or by win-stay/lose-shift behavior.

Signal	Brain regions	MNI coordinates (mm)			z value
		x	y	z	
Outcome event					
	Brain stem	4	-29	-17.5	12.7
	Ventral premotor cortex	24.5	-6.5	7.5	11.9
	lOFC	22	8	-2.5	10.7
	Ventral V4	25	-31.5	-5.5	9.78
	Area TEO	-22	-26	-7	7.95
	lOFC	-20.5	8	-2.5	8.65
	Ventral V2	24.5	-42.5	-4.5	8.75
	Brain stem	3	-30.5	-26	8.96
	V1	19	-47.5	-2.5	7.73
	Ventromedial prefrontal cortex	0	12.5	-1	8.17
	V1	0	-46	8	8.37
	Cerebellum	-6.5	-26.5	-15	8.25
	Ventromedial prefrontal cortex/frontal pole	0.5	23.5	0	9.9
	Cerebellum	20	-37.5	-10	9.5
	Cerebellum	-12.5	-40.5	-15.5	7.25
Win-stay/lose-shift					
	lOFC	17.5	9.5	-4	3.67
	Dorsal V4/area 7a	-23	-26	12	4.19

Supplemental Experimental Procedures

Behavioral Training

Prior to actual data acquisition, all animals were trained to work in an MRI compatible chair in a sphinx position that was placed inside a custom mock scanner that simulated the MRI scanning environment. They were trained to use custom-made infra-red touch sensors to respond to stimuli presented on a screen. They learned to perform the ODR task (sometimes called a stimulus reversal task). The mock scanner training was considered complete once the animals performed with greater than 75% accuracy for at least three consecutive training sessions. The animals underwent aseptic surgery to implant an MRI compatible head post (Rogue Research, Mtl, CA). After a recovery period of at least two months, the animals were trained to perform the task inside the actual MRI scanner under head fixation. The imaging data acquisition started once they performed at above 75% accuracy for at least another three consecutive sessions in the scanner.

FMRI data preprocessing

FMRI data were corrected for body motion artefact by an offline-SENSE reconstruction method (Kolster et al., 2009) (Offline_SENSE GUI, Windmiller Kolster Scientific, Fresno, CA). The images were aligned to an EPI reference image slice-by-slice to account for body motion and then aligned to subject’s structural volume to account for static field distortion (Kolster et al., 2014) (Align_EPI GUI and Align_Anatomy GUI, Windmiller Kolster Scientific, Fresno, CA). The aligned data were processed with high-pass temporal filtering (3-dB cutoff of 100s) and Gaussian spatial smoothing (full-width half maximum of 3mm). The data that were already registered to subject’s structural space was registered to the CARET macaque F99 template using affine transformation.

Time course analysis

We analyzed activity time courses by extracting the signal size of the peaks from individual testing sessions. In particular, a window for extraction was established by the full-width half-maximum of each peak tested. The exact time point for signal extraction for a given session was determined by the position of the group peak using a leave-one-out approach to avoid bias. In order to test the significance of a peak, the extracted signal sizes were averaged within subject, cube-root transformed to account for the skewness of the data and then tested using a one-sample t test (two-tailed).

We examined the relationship between the session-to-session variation in BOLD signal sizes and session-to-session variation in behavior. In order to remove between subject variance and to spare between session variance within the same subject each extracted neural signal size was normalized by subtracting the mean signal size across sessions within the same subject and divided by the standard deviation of the signal sizes for that subject. A similar normalization procedure was applied to behavioral indices: each session's behavioral index was normalized by subtracting the mean behavioral index across sessions for a given subject and then dividing by the standard deviation of the behavioral indices for that subject. Note that the normalization procedure is only used in the analyses examining the relationship between individual variation in neural activity and individual variation in behavior. It cannot be used prior to calculating each individual's mean effects in the initial test of the significance of the group neural effect because it will reduce all effects to zero.

Reinforcement learning model

In Figure 2, we show that variation in lOFC signal strength was related to variation in accuracy in the learning phase of the task and not to variation in accuracy in the post-learning phase of the task. Variations in accuracy in the initial learning phase of the task and in the post-learning phase of the task probably reflect, respectively, variation in learning rate and choice stochasticity respectively. In Figure S5 we provided additional analyses confirming the different relationships between lOFC signals and behavior by using learning rates and temperature parameters estimated by a computational model. The learning rate parameter α was estimated by the Rescorla-Wagner model (Rescorla and Wagner, 1972):

$$V_{t+1,s} = \begin{cases} V_{t,s} + \alpha\left(r_t - V_{t,s}\right), & \text{if option } s \text{ was chosen} \\ V_{t,s}, & \text{if option } s \text{ was unchosen} \end{cases}$$

where $V_{t,s}$ and r_t are the value of option *s* and the choice outcome on trial *t* respectively. α is a learning rate free parameter.

The stochasticity parameter was estimated by applying a softmax function that models probabilities of choosing each option:

$$P_{t,s} = \frac{\exp(V_{t,s}/T)}{\sum_{s'=1}^{2} \exp(V_{t,s'}/T)}$$

where $P_{t,s}$ is the probability of choosing option *s* on trial *t*. *T* is a temperature free parameter describing the degree of stochasticity.

The free parameters *α* and *T* from the Rescorla-Wagner model and the softmax function respectively were fitted session-by-session by minimizing the negative log likelihood *L*:

$$L = -\sum_{t=1}^{N} \log(P_{t,c_t})$$

where *N* is the total number of trials and c_t is monkey's choice on trial *t*.

Supplemental References

Belova, M.A., Paton, J.J., Morrison, S.E., and Salzman, C.D. (2007). Expectation modulates neural responses to pleasant and aversive stimuli in primate amygdala. Neuron *55*, 970-984.

Grill-Spector, K., Henson, R., and Martin, A. (2006). Repetition and the brain: neural models of stimulus-specific effects. Trends Cogn Sci *10*, 14-23.

Kolster, H., Janssens, T., Orban, G.A., and Vanduffel, W. (2014). The Retinotopic Organization of Macaque Occipitotemporal Cortex Anterior to V4 and Caudoventral to the Middle Temporal (MT) Cluster. J Neurosci *34*, 10168-10191.

Kolster, H., Mandeville, J.B., Arsenault, J.T., Ekstrom, L.B., Wald, L.L., and Vanduffel, W. (2009). Visual field map clusters in macaque extrastriate visual cortex. J Neurosci *29*, 7031-7039.

Ousdal, O.T., Specht, K., Server, A., Andreassen, O.A., Dolan, R.J., and Jensen, J. (2014). The human amygdala encodes value and space during decision making. Neuroimage *101*, 712-719.

Peck, C.J., Lau, B., and Salzman, C.D. (2013). The primate amygdala combines information about space and value. Nat Neurosci *16*, 340-348.

Rescorla, R., and Wagner, A. (1972). A theory of Pavlovian conditioning: variations in the effectiveness of reinforcement and nonreinforcement. In Classical Conditioning, A.H. Black, and W.F. Prokasy, eds. (New York: Appleton-Century-Crofts).

Sutton, R.S., and Barto, A.G. (1998). Reinforcement learning : an introduction (Cambridge, Mass.: MIT Press).
